# On the Brink: Mapping the Last Strongholds of the Critically Endangered Flapper Skate (*Dipturus intermedius*)

**DOI:** 10.1002/ece3.71650

**Published:** 2025-06-30

**Authors:** Sophie L. Loca, Patrick C. Collins, Amy Garbett, Ryan McGeady, James Thorburn, Chris McGonigle

**Affiliations:** ^1^ School of Biological Sciences Queen's University Belfast Belfast UK; ^2^ School of Geography and Environmental Sciences Ulster University Coleraine UK; ^3^ Centre for Conservation and Restoration Science, School of Applied Sciences Edinburgh Napier University Edinburgh UK

**Keywords:** Bayesian, common skate, conservation, Dipturus, fisheries, INLA

## Abstract

The flapper skate (*Dipturus intermedius*) is a Critically Endangered skate distributed throughout the NE Atlantic and requiring urgent conservation measures. Existing models of the flapper skate's distribution are not detailed enough to inform management. The aim of this study was to develop more highly resolved predictions of the skate's distribution across its range, building on existing studies to provide a comprehensive baseline for flapper skate presence. Location The NE Atlantic shelf region. A Bayesian spatial binomial GAMM was used to model the distribution of flapper skate across the NE Atlantic shelf. Following an exhaustive search of fisheries‐independent DATRAS catch records, skate presence was modelled as a function of environmental covariates and AIS fishing pressure data. Skate presence was highest in coastal areas approximately 40–50 km from shore, where fishing pressure and benthic productivity were low. A smoother for the bathymetry variable indicated presence was highest at depths of 100–200 m. Regions with the highest predicted probability of occurrence included the north and west coasts of Scotland, and the west coast of Ireland near Counties Clare and Galway. In contrast, very low support was given for presence in the southern and central North Sea, likely reflecting historical population collapse, as well as in deeper offshore waters beyond the shelf. This study presents the first large‐scale model of flapper skate presence across the NE Atlantic shelf that integrates both environmental and fishing pressure data, providing new baseline insights into habitat use in the North Sea and around Ireland. Three core regions of presence were identified, likely reflecting natural refugia from fishing and critical habitats (EFHs). Future research should prioritise these strongholds, focusing on identifying critical habitats to support focused management strategies.

## Graphical abstract

This study presents a high‐resolution spatial distribution model for the Critically Endangered flapper skate (*Dipturus intermedius*) across the NE Atlantic shelf, integrating environmental and fishing pressure data. Results highlight three core regions of presence off the coasts of Scotland and western Ireland, likely reflecting natural refugia and the presence of essential habitats. These findings provide a critical baseline to inform targeted conservation and spatial management strategies.

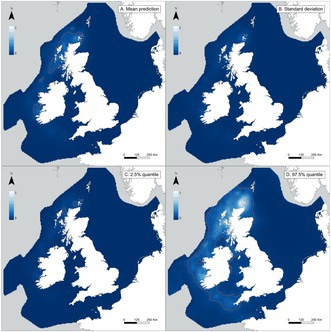

## 1 Introduction

The flapper skate (*Dipturus intermedius*) is a large‐bodied, Critically Endangered elasmobranch, distributed from Northern Norway (63° N) to as far south as the Azores (Ellis et al. 2021; Garbett et al. 2023). Although historically of little commercial value, the species is a popular amongst recreational anglers and divers in the UK, which is a source of income and considerable monitoring data for the species (Kenter et al. 2013; Neat et al. 2015; Régnier et al. 2024). Like other large‐bodied elasmobranchs, it follows a k‐selected life history strategy, characterised by slow growth, late maturity and low reproductive output (Brander 1981; Dulvy et al. 2000; Thorburn et al. 2023; Régnier et al. 2024). As a result, the flapper skate is considered highly susceptible to any form of exploitation or impacts on recruitment (Brander 1981; Dulvy et al. 2000; Dulvy and Reynolds 2002; Régnier et al. 2021). This vulnerability, combined with sustained overfishing throughout the 20th century, led to severe population declines and local extirpations throughout the NE Atlantic (Brander 1981; Dulvy et al. 2000; Dulvy and Reynolds 2002; Ellis et al. 2021), such that the flapper skate is now a threatened species of global conservation concern (NIEA 2004; HELCOM 2006; OSPAR Commission 2008; NatureScot 2020; Ellis et al. 2021).

Due to a high degree of morphological conservatism shared by the long‐nosed skates (genus *Dipturus*; Iglésias et al. 2010), the flapper skate has been the subject of several taxonomic revisions (Garbett et al. 2023). Previously grouped with the common blue skate (now *D. batis* ) under the ‘common skate’ (then *D. batis* ) nomenclature, the flapper skate received formal recognition as a species in 2021 (Ellis et al. 2021), following molecular and morphological evidence published a decade earlier (Griffiths et al. 2010; Iglésias et al. 2010). Although superficially similar, the two species have distinct life histories and ecologies, which necessitate tailored management strategies (Ellis et al. 2021; Garbett et al. 2023). For example, the larger body size and later age at maturity of the flapper skate (Iglésias et al. 2010), means that it is more vulnerable to depletion than the blue skate (Dulvy and Reynolds 2002). This longstanding taxonomic confusion has diminished the relevance of most available data for the flapper skate, and ongoing misidentification of these species has affected data quality even where the correct nomenclature is used (Iglésias et al. 2010; Ellis et al. 2021; Garbett et al. 2021). Therefore, our present knowledge base for the species is extremely limited and several gaps exist around its ecology, distribution and genetics (Garbett et al. 2021).

Given the vulnerability of the flapper skate to fishing pressure (Walker and Hislop 1998; Dulvy and Reynolds 2002; Dulvy et al. 2021), an understanding of where they are most vulnerable is essential for effective management. Although previous investigations utilising recreational angling records, genetic samples and fisheries‐independent data have painted a broad picture of the flapper skate's presence and absence across its range (Lynghammar et al. 2014; Frost et al. 2020; Garbett et al. 2023), further research is required to better understand the flapper skate's presence within these areas. Species distribution models (SDMs) allow researchers to link occurrence records with environmental data (Guisan and Zimmermann 2000) to provide insight into a species' habitat use (Guisan et al. 2013; Laman et al. 2018) and environmental sensitivities (Logez et al. 2012). The model outputs can also indicate where they are most exposed to fisheries pressures (Cosandey‐Godin et al. 2015; Mannocci et al. 2020; Jubinville et al. 2022) and are therefore useful for informing conservation measures or signposting future research efforts to do so (Guisan et al. 2013; Jubinville et al. 2021).

To date, three studies have modelled the distribution of the flapper skate (Pinto et al. 2016; Bache‐Jeffreys et al. 2021; Régnier et al. 2024). Pinto et al. (2016) first modelled skate presence off the west coast of Scotland using fisheries‐independent survey records and validated the findings with animal tracking data. This study found depth and distance to coast to be key variables predicting species presence, with skate occupying largely inshore waters and depths up to 300 m (Pinto et al. 2016). More recently, skate presence in this region was modelled a second time with fisheries‐independent catch data, as part of a wider study on recovery rates within the Loch Sunart to the Sound of Jura Marine Protected Area (LSSJ‐MPA; Régnier et al. 2024). They found skate presence was highest at depths of 150–200 m and habitats with a higher proportion of sand substrate (Régnier et al. 2024).

Bache‐Jeffreys et al. (2021) was the first to model the flapper skate's distribution across the NE Atlantic shelf using georeferenced genetic records (*n* = 27). This study was successful in predicting skate presence across its range and revealed surface and benthic productivity and bottom temperature to be key drivers of skate presence (Bache‐Jeffreys et al. 2021). Broad regions of importance to skate were highlighted, including the west coast of Ireland and Scotland, the south coast of Iceland and the North Sea (Bache‐Jeffreys et al. 2021). Although the use of molecular techniques improved the reliability of their data compared to Pinto et al. (2016) and Régnier et al. (2024), the low sample size in Bache‐Jeffrey et al.'s study precluded robust predictions of sufficient detail (Bache‐Jeffreys et al. 2021). To model skate habitat use across such a wide study area, a larger dataset incorporating absence information would improve the accuracy and resolution of predictions (Brotons et al. 2004; Hanberry et al. 2012; Grimmett et al. 2020).

Fisheries‐independent surveys provide a practical solution to address the need for large‐scale, standardised presence–absence data. Due to its large body size, the flapper skate is vulnerable to incidental capture throughout its life, resulting in opportunistic catch records across much of its range (Iglésias et al. 2010; Pinto et al. 2016; Frost et al. 2020; Garbett et al. 2023). Stock assessment surveys tend to be conducted annually using consistent methodologies across broad geographic areas, making them particularly valuable for species distribution modelling (Maes et al. 2015; Petersen et al. 2021). Subsequently, these data have been widely applied in models across marine systems (Munoz et al. 2013; Pennino et al. 2013; Cosandey‐Godin et al. 2015; Maes et al. 2015; Paradinas et al. 2015; Paradinas et al. 2016; Laman et al. 2018; Lezama‐Ochoa et al. 2020; Moriarty et al. 2020; McGeady et al. 2022; Elliott et al. 2023; Howard et al. 2023). However, variations in gear use, as well as biases in survey design and reportage, can result in statistical biases which can impact model performance (Martínez‐Minaya et al. 2018; Zeller and Pauly 2018).

To improve the reliability of SDM outputs, statistical artefacts can be accounted for by the inclusion of additional data or modelling terms (Martínez‐Minaya et al. 2018; Moriarty et al. 2020). The Integrated Nested Laplace Approximation (INLA) is a flexible Bayesian modelling environment which easily incorporates spatial, temporal and random effects (Rue et al. 2009, 2017; Beguin et al. 2012; Redding et al. 2017; Martínez‐Minaya et al. 2018). This flexibility enables users to address complex non‐linear interactions, spatial autocorrelation and high dimensionality (Rue et al. 2017; Martínez‐Minaya et al. 2018). As a result, this framework has seen increasing popularity since its conception (Rue et al. 2017) and has been applied to investigate fisheries bycatch (Cosandey‐Godin et al. 2015), sensitive habitats (Pennino et al. 2013; Paradinas et al. 2015) and species occurrences (Munoz et al. 2013; Knapp et al. 2016; Paradinas et al. 2016; Lezama‐Ochoa et al. 2020).

Employing the INLA modelling framework, this study aimed to generate the first high‐resolution, spatially explicit predictions of flapper skate presence across the NE Atlantic shelf, to support the conservation efforts for the species. To capture the contemporary distribution of the species, fisheries‐independent data from 2010 to 2023 were extracted for the entire study area.

## 2 Methods

### 2.1 Occurrence Data

**FIGURE 1:**
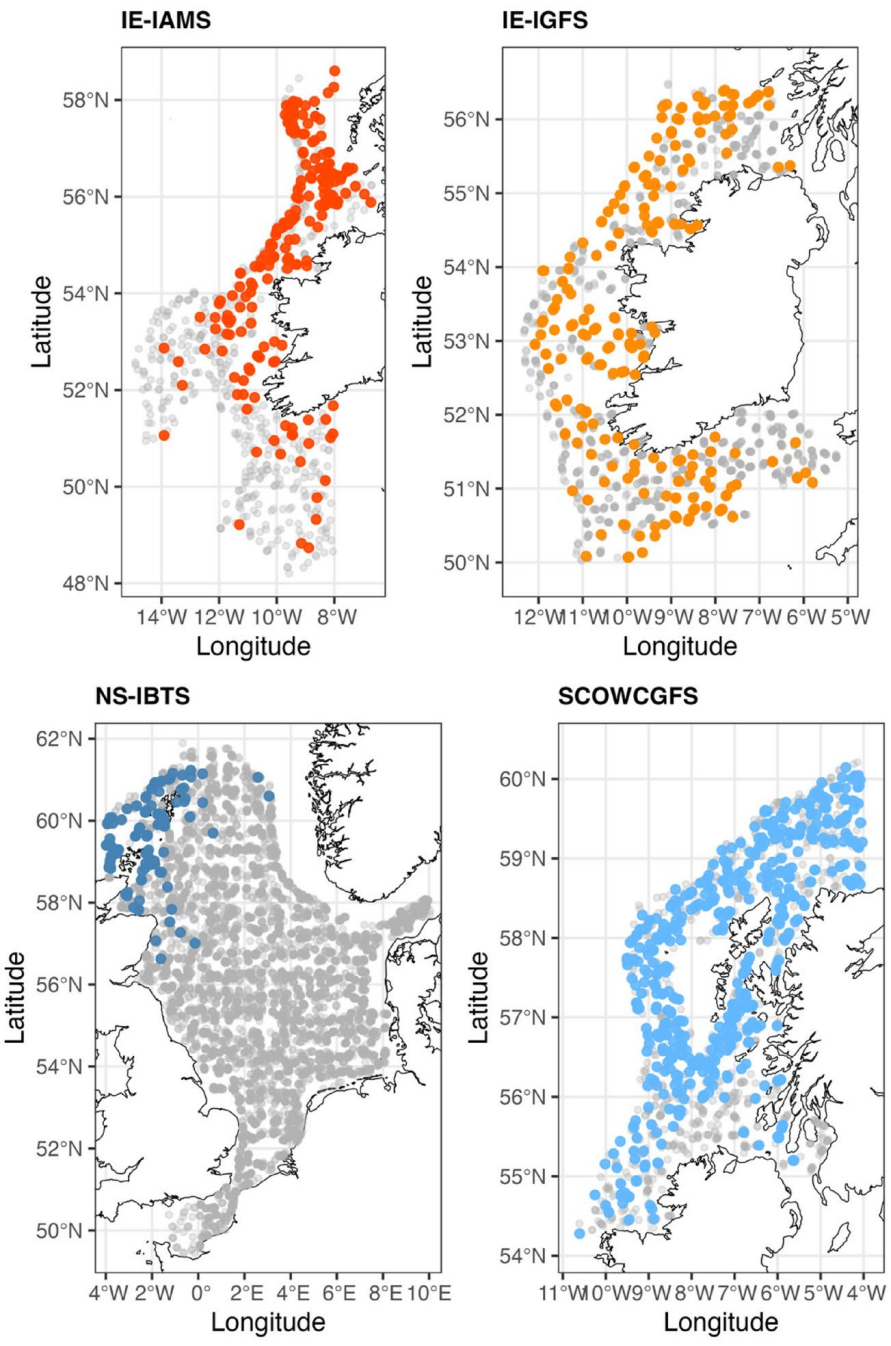
Maps of species occurrence data used to model the distribution of flapper skate in the NE Atlantic. Data were obtained from the ICES DATRAS database for four surveys conducted 2010–2023: The Irish Anglerfish and Megrim Survey (IE‐IAMS), Irish Groundfish Survey (IE‐IGFS), North Sea International Bottom Trawl Survey (NS‐IBTS), and Scottish West Coast Groundfish Survey (SCOWCGFS). Coloured points represent hauls where the species was present; grey semi‐transparent points denote absences.

Flapper skate catch records were obtained from ICES DATRAS hereafter as ‘DATRAS’, the Database of Trawl Surveys managed by International Council for the Exploration of the Sea (ICES DATRAS 2023). ‘HH’ haul data were extracted for the surveys within the NE Atlantic shelf region for the years ‘2010–2023’ (*n* = 19), using the ‘icesDATRAS’ package in R (Millar et al. 2017; see Figure S1). HL ‘length‐based’ catch records were also extracted for the flapper skate (species code: 711846) for each survey (Table 1). Only those surveys with an adequate number of presence records for skate were retained for further analysis: the Irish Anglerfish and Megrim Survey (IE‐IAMS), Irish Groundfish Survey (IE‐IGFS), the North Sea International Bottom Trawl Survey (NS‐IBTS) and the Scottish West Coast Groundfish Survey (SCOWCGFS). For these datasets, flapper skate catch records were aggregated to produce presence or absence per haul (Figure 1) and then combined into one occurrence data set (*n* = 14,345).

**TABLE 1:** Summary of fisheries‐independent survey data available from the DATRAS database, for the years 2010–2023 and the NE Atlantic shelf region.

Code	Survey name	Years	Quarters	Total haul number	Hauls with *D. intermedius* present	Hauls with *D. intermedius* absent
BITS	Baltic International Trawl Survey	2010–2023	1, 4	8461	0 (0.00%)	8461
BTS	Beam Trawl Survey	2010–2023	1, 3, 4	9720	1 (0.01%)	9719
BTS‐VIII	Beam Trawl Survey—Bay of Biscay (VIII)	2011–2022	4	695	0 (0.00%)	695
FR‐CGFS	French Channel Ground Fish Survey	2010–2022	4	1036	0 (0.00%)	1036
EVHOE	French Southern Atlantic Bottom Trawl Survey	2010–2022	4	1796	5 (0.27%)	1791
FR‐WCGFS	French Western English Channel Ground Fish Survey	2021	3	50	0 (0.00%)	50
DYFS	Inshore Beam Trawl Survey	2010–2023	3, 4	7841	0 (0.00%)	7841
IE‐IAMS	Irish Anglerfish and Megrim Survey	2016–2022	1, 2	735	206 (28.02%)	529
IE‐IGFS	Irish Groundfish Survey	2010–2022	1	2071	274 (13.23%)	1797
NL‐BSAS	Netherlands Industry Survey on Turbot and Brill	2019–2023	3, 4	290	0 (0.00%)	290
NS‐IBTS	North Sea International Bottom Trawl Survey	2010–2023	1, 3	10,063	141 (1.40%)	9922
NSSS	North Sea Sandeel Survey	2010–2023	4	4317	0 (0.00%)	4317
NIGFS	Northern Ireland Groundfish Survey	2010–2022	1, 4	1541	0 (0.00%)	1541
NS‐IDPS	Norwegian Sea International Deep Pelagic Survey	2012–2016	1, 3	183	0 (0.00%)	183
SCOROC	Scottish Rockall Survey	2011–2023	3	566	5 (0.88%)	561
SWC‐IBTS	Scottish West Coast Bottom Trawl Survey	2010	1	61	0 (0.00%)	61
SCOWCGFS	Scottish West Coast Groundfish Survey	2011–2023	1, 4	1476	555 (37.60%)	921
SNS	Sole Net Survey	2010–2023	3, 4	653	0 (0.00%)	653
SE‐SOUND	Sweden Sound Survey	2011–2022	1, 3, 4	135	0 (0.00%)	135

*Note:* Percentages in brackets are the proportion of total hauls in the survey where *D. intermedius* was present. Shaded rows represent the datasets considered for species distribution analysis.

Although it is possible to filter for adult flapper skate based on total length measurements (Iglésias et al. 2010; see Garbett et al. 2023), it was not possible to implement this filter in the present study while retaining sufficient data for the analysis. Therefore, hereon in, *D. intermedius* records were considered valid records while acknowledging the caveats with these data.

### 2.2 Environmental Data

Environmental predictor variables were selected based on established models of skate distribution and included depth (m), distance from coast (m), mean bottom temperature (°C), mean bottom current velocity (ms^−1^), mean maximum benthic primary productivity (mmol. m‐3) and mean monthly fishing pressure (hours per month; Table 2). Substrate type was considered as a potential explanatory variable, but due to inconsistent coverage and significant data gaps across the full study area, it was excluded from the present analysis. Depth was obtained from the General Bathymetric Chart of the Oceans (GEBCO; GEBCO Compilation Group 2020). Monthly averages of bottom temperature, northward current velocity and eastward current velocity were obtained from the EU Copernicus Marine Service Information as raster files (Tonani and Ascione 2021). Since a dataset for bottom current velocity was not available, the velocity at the seafloor was calculated using a bathymetry mask (see Bina 2023). Fishing pressure data were obtained from Global Fishing Watch (Global Fishing Watch 2025a) and represented ‘apparent fishing pressure’ modelled using Automatic Identification System (AIS) data for commercial fishing vessels. Monthly average datasets were extracted by flag State and vessel class for the years 2012–2023. The data were then filtered to include only the following gear types: ‘trawlers,’ ‘fixed_gear,’ ‘fishing,’ ‘set_gillnets,’ ‘dredge_fishing,’ ‘trollers,’ and averaged across years and cells to produce a single raster file. A raster for benthic primary productivity at maximum depth was downloaded from bio‐oracle (Tyberghein et al. 2012; Assis et al. 2024) for the years 2010–2020 and represented mean values averaged for the decade. A coastline shapefile was obtained from the European Environment Agency (European Environment Agency 2015).

**TABLE 2:** Sources of environmental predictor variables used to model flapper skate presence in the NE Atlantic shelf region.

Variable	Source	Spatial extent (x^1^, x^2^, y^1^, y^2^)	Resolution	Temporal extent
Bathymetry (m)	EMODnet	NE Atlantic (−20, 15, 40, 65)	0.001° x 0.001	—
Benthic mean primary productivity – maximum depth (mmol·m^−3^)	Bio‐ORACLE	Global (−180, 180, −90, 90)	0.05° x 0.05°	Decadal mean 2010–2020
Bottom temp (°c)	Copernicus Marine Service	NE Atlantic (−20, 13, 40, 65)	0.111° × 0.067°	Monthly mean 2010–2022
Distance to coast (m)	Calculated in QGIS	—	—	—
Eastward and northward current velocity (ms^−1^)	Copernicus Marine Service	NE Atlantic (−20, 13, 40, 65)	0.111° × 0.067°	Monthly mean 2010–2022
Fishing pressure (hours per month)	Global Fishing Watch	NE Atlantic (−16, 11, −47, 63)	0.01° x 0.01°	Monthly mean 2012–2023

*Note:* The data were processed in ArcGIS Pro and extracted for each location point in R studio.

Bottom temperature and bottom current velocity were extracted for each year covered by the occurrence data and combined into raster stacks. The mean monthly averages of each variable across these time periods were generated by using the ‘calc’ function from the raster package (version 3.1–5, Hijmans 2021). Northward and eastward bottom current velocity were combined to form a combined current speed using the ‘uv2ds’ function from the rWind package (version 1.0.4, Fernández‐López and Schliep 2019). Primary productivity data represented one average across the time series, so no additional processing was required. The environmental data corresponding to each occurrence point were then extracted from the raster layers and joined to create a combined dataset of occurrences and habitat variables. Extractions were carried out using the ‘extract’ function as part of the raster package. Distance from coast was calculated for each occurrence point using the ‘Join attributes by nearest’ tool in QGIS. Data points that contained null values for environmental variables were dropped, resulting in a total sample size of 13,192.

### 2.3 Data Exploration

Initial data exploration was carried out according to methods outlined by Zuur et al. (2010); predictor variables were checked for collinearity, outliers and normality. Correlations between variables were observed within a correlation matrix generated using the corrplot package (Wei and Simko 2021; version 0.92), where pairs with *r* > 0.6 were found, one of the variables was removed (Zuur et al. 2017). Similarly, variables with a high variance inflation (Generalised Variance Inflation Factor > 3) were identified and removed (Zuur et al. 2010, 2017). Following the collinearity checks, bottom temperature was dropped from the analysis (see Table S1 and Figure S2). Outliers were observed using boxplots, and offending data points were investigated for error. Records at depths greater than 1000 m (*n* = 18), with greater than 80 min haul duration (*n* = 1) and with fishing hours over 100 (*n* = 22) were removed. Normality and skewness of the data were checked by plotting histograms of each covariate using the ‘ggplot’ function from the ggplot2 package (Version 3.3.5, Wickham 2016). After processing, the dataset contained a total of 13,151, with 1174 (8.93%) presence records and 11,977 (91.07%) absence records.

Environmental variables were compared between presence and absence hauls visually with boxplots and empirically with Wilcoxon rank sum tests (stats package version 3.6.2, R Core Team 2023). Continuous predictor variables were standardised before modelling to avoid numerical estimation problems and to improve interpretation of the regression parameters (Zuur et al. 2017).

### 2.4 INLA

The INLA framework (Rue et al. 2009) was used to model flapper skate presence in the NE Atlantic in R (www.r‐inla.org/). INLA is designed to work with latent Gaussian models, a class of models that includes generalised linear models, spatial and spatio‐temporal models (Rue et al. 2017). This framework employs the Stochastic Partial Differential Equations (SPDE) approach to capture spatial effects in the model (Lindgren et al. 2011). To simplify calculations, a Gaussian Markov random field (GMRF; Rue and Held 2005) is created, where only neighbourhood sites have non‐zero covariance values (Zuur et al. 2017). This replaces the traditional spatio‐temporal covariance function and improves computation times drastically (Cameletti et al. 2013; Zuur et al. 2017). The SPDE function uses a finite element representation to define the Matérn spatial random field, with linear basis functions defined on a triangulation of the domain (Cameletti et al. 2013). This sparse, triangulated ‘mesh’ replaces the continuously indexed random field, and links the Gaussian field and the GMRF, to which a Markovian structure can be given. INLA then calculates the posterior conditional distribution of the GMRF for each of the mesh's vertices, and once given the prediction of y at that location is immediate (Cameletti et al. 2013). The spatial effect links observations and their spatial locations, thus accounting for regions of noise in the data that cannot be explained by the available covariates (Munoz et al. 2013).

### 2.5 Model Development

To develop a robust model, a stepwise approach was taken to incorporate key dependencies. Temporal dependency was accounted for by applying a rw1 random walk trend to the year and quarter (time of year) variables (Zuur et al. 2017). To account for survey‐related changes in catchability, survey was modelled as an independent and identically distributed (iid) random effect, and haul duration was modelled with a second‐order random walk (rw2) trend. The remaining environmental variables were included as fixed effects. This formulation served as the model's initial structure (model A).

To assess the need for additional smoothing terms, the non‐linear effects of environmental covariates in model A were explored (following Zuur et al. 2017). A series of GAMs were applied on the residuals of this initial model with each of the covariates as smoothers, using the ‘gam’ function from the mgcv package (Version 1.8–36, S. Wood 2021). The Akaike Information Criterion (AIC) was used to assess which of the covariates improved the model when smoothed (Bozdogan 1987). Covariates that lowered the AIC value were included as smoothers and modelled using a rw2 smoother within the INLA package (model B).

To capture spatial effects, a SPDE mesh was constructed using the ‘inla.nonconvex.hull’ function to define a convex hull boundary around the data points (Figure S3). With the boundary as an argument, a mesh was generated using the ‘inla.mesh.2d’ function. The SPDE term was added to the previous formulation to form a spatial model with environmental smoothers (model C).

A final model was constructed to assess the impact of the link function. The choice of link function is critical in statistical modelling, as it defines how predictors relate to the response variable (Damisa et al. 2017). While the logit link is commonly used for binary outcomes, the complementary log–log (cloglog) link is often preferable when event probabilities are highly skewed (Piegorsch 1992). To evaluate the choice of link function, model D incorporated the cloglog link in addition to environmental smoothers and the spatial effect, allowing for a comparison of model performance against previous formulations.

### 2.6 Modelling Presence of *D. intermedius*

The binomial model was specified as follows: *D. intermedius* presence or absence at a location *i* (*i* = 1,…, *n*, *n* = sample size) = y_i_. y_i_ was = 0 if absent and y_i_ = 1 if present. We assumed y_*i*_ ~ Bernoulli(π_i_), where π_ti_ is the probability of presence of *D. intermedius* at location *i* and year *t*. Models were then defined as:

(1) $$\text{cloglog}\left(\pi_{\mathrm{ti}}\right)=\alpha_{0}+X_{i}\beta +W_{i}$$

where α_0_ is the intercept, β is the vector of regression parameters, X_*ti*_ is the explanatory covariate matrix at location *i* and year *t* and W_*i*_ represents the spatial random effects at *i* (Lezama‐Ochoa et al. 2020). The modelling process was conducted in five steps as follows: (1) construct a triangulated mesh, (2) build a SPDE, (3) create a stack for the data, (4) specify the model and (5) run INLA for modelling and estimation (Zuur et al. 2017).

### 2.7 Model Selection

The final model formulation was chosen based on the following criteria, the corrected Akaike information criterion (AICc; Hurvich and Tsai 1989), the Bayesian information criterion (BIC; Schwarz 1978), the deviance information criterion (DIC; Spiegelhalter et al. 2002) and the Watanabe–Akaike information criterion (WAIC; Watanabe 2010). A cross‐validation analysis was also used to inform model selection. The recently developed leave‐group‐out cross‐validation (LGOCV; Liu and Rue 2022; Adin et al. 2024) is a robust measure of predictive performance for models where space and time dependencies are present. This technique works by partitioning the data into meaningful groups (such as spatial or temporal groups), which are iteratively ‘left out’ for testing while the remaining data is used to train the model (Liu and Rue 2022; Adin et al. 2024). The analysis was carried out using the ‘inla.group.cv’ function in R‐INLA and groups were constructed automatically based on posterior correlations between the linear predictors (Liu 2023). For each iteration (*n* = sample size), the log‐transformed cross‐validation (CV) scores were calculated. The mean log CV score and mean square predictive error (MSPE) was calculated for each model (see Adin et al. 2024), with lower values indicating better predictive performance. These LGOCV metrics were used to compare model predictive performance. Environmental covariate selection was not undertaken.

### 2.8 Model Prior Specification

In Bayesian statistics, priors represent initial beliefs about a parameter before observing data (Gelman et al. 2013). In INLA models, they help regularise estimates and prevent overfitting, particularly with limited data or complex models (Zuur et al. 2017). For *Dipturus intermedius*, prior information was either unavailable or inapplicable. Although Pinto et al. (2016) found that skate presence declined beyond 200 m depth, this pattern may have been influenced by the unique seabed topology in their study area. As a result, depth was not used as prior information across the species' range.

Instead, weakly informative priors were used to stabilize fixed effect estimations and to avoid imposing strong assumptions on the model (Northrup and Gerber 2018). Following Northrup and Gerber (2018), an initial prior was specified with variance approximately equal to 2 (standard deviation ≈ 1.4). Alternative priors with gradually smaller standard deviations (SD = 1.2, SD = 1, SD = 0.8 and SD = 0.5) were also tested to investigate the sensitivity of the model to prior choice. The prior sensitivity test was conducted on the final model and showed that estimates were generally robust to changes in prior choice. Estimates for current speed and fishing hours remained stable across priors, while the intercept and longitude showed greater sensitivity (Figure S4).

### 2.9 Model Validation

Validation of the final model was formally evaluated with the Area Under the Curve (AUC), Sensitivity (true positive rate, TPR) and Specificity (true negative rate, TNR). A classification threshold was chosen using the ‘SSS’ method (Jiménez‐Valverde and Lobo 2007; Liu et al. 2013), which maximised the sum of sensitivity and specificity. Following a comparison of several thresholds, a value of 0.1 was chosen (see Table S2 for full set of thresholds tested), which matched the prevalence of the dataset.

Receiver Operator Curves (ROC) evaluate a model's classification performance by plotting the TPR vs. the false positive rate (FPR; Hanley and McNeil 1982) and are a widely adopted measure of model predictive performance. However, they can be very sensitive to low prevalence datasets with a large biogeographical extent, within which true absences are inflated (Sofaer et al. 2019). For this reason, a Precision‐Recall (PR) curve, representing Precision (Positive Predictive Value, PPV) vs. Recall (TPR; Davis and Goadrich 2006), was also constructed. Since they do not incorporate true negatives into their calculations, PR‐curves are a more robust measure of performance for imbalanced, or low prevalence data (Davis and Goadrich 2006; Sofaer et al. 2019). Higher AUC values for ROC and PR‐curves indicate better class discrimination (i.e., presence vs. absence) and better precision‐recall trade‐offs, respectively. For ROC, values closer to 1 indicate better predictive performance and values near 0.5 suggest no better than random (Fielding and Bell 1997). For low‐prevalence datasets, PR‐AUC values of at least 0.5 are considered good (see Sofaer et al. 2019). ROC and PR‐curves were generated using the ‘roc.curve’ and ‘pr.curve’ functions, respectively, from the PRROC package (version 1.3.1, Grau and Keilwagen 2018).

To independently test the predictive power of the model, a simulation study was performed according to Zuur et al. (2017). The ‘inla.posterior.sample’ function in R‐INLA was used to simulate parameters from the posterior distributions. This was repeated to create 1000 sets of parameters from which fitted values were generated. The ‘rpois’ function was then used to simulate skate presence–absence from the fitted values, which is also part of the r‐INLA package. The predictive ability of the model was tested by comparing the proportion of zeros produced from the simulated datasets with the observed data, using the 0.1 classifier threshold (Zuur and Ieno 2016; Zuur et al. 2017). The agreement between observed and simulated counts was assessed using a Z‐score, where values within ±1.96 standard deviations indicate a close match and good model predictive performance. Finally, the dispersion statistic (e.g., the sum of squared Pearson residuals) of each model was calculated: a value around 1 indicated that the data were not over‐ or under‐dispersed (Zuur et al. 2017). A semi‐variogram was also plotted to assess the spatial dependence in the residuals of the model, following Zuur et al. (2017).

### 2.10 Predicting the Occurrence of *D. intermedius*

Predictions of the posterior mean and standard deviation of probability of presence of *D. intermedius* were generated within the INLA function by including an empty prediction stack and a data frame containing location points and associated environmental data. The location points were generated by using the ‘Create grid’ tool in QGIS with a resolution of 10 km, using the occurrence data as the grid extent. Environmental data were extracted for these points as previously described, and null values were removed where the grid overlapped with land, resulting in a total of 11,138 locations. The probability of presence was generated for each of these points and extracted. The spatial random field of each model was plotted by using the ‘inla.mesh.project’ function in INLA and converted into a data frame. Prediction and random field maps were then created in Arc Pro using the ‘Empirical Bayesian Kriging’ tool from the Geostatistical Analyst Tools set to create a raster within the extent of the study area.

## 3 Results

Pairwise comparisons of environmental variables between presence and absence locations showed significant differences for all variables (Figure S5).

Model D showed the best overall performance, with the lowest DIC, WAIC, AICc and log mean cross‐validation score (Table 3). Despite having a higher BIC value due to its complexity, model D was selected to provide balance between fit and predictive accuracy. The final model formulation included a smoother for distance to coast, a spatial SPDE term, and a cloglog link.

$$\begin{array}{c} \text{Skate}01\sim \text{Intercept}+\text{Benthic Primary Productivity}\\ \;+\text{Bottom Current Speed}+\text{Distance to Coast}\\ \;+\text{Fishing Pressure}\;\left(\text{hours}\;\mathrm{per}\;\text{month}\right)\\ \;+\text{Longitude}\;\left(\mathrm{xkm}\right)+\text{Latitude}\;\left(\mathrm{ykm}\right)+f\left(\text{Bathymetry},\mathrm{Rw}1\right)\\ \;+f\left(\text{Haul Duration},\mathrm{Rw}2\right)+f\left(\text{Year},\mathrm{Rw}1\right)\\ \;+\left(\text{Quarter},\mathrm{iid}\right)+\left(\text{Survey},\mathrm{iid}\right)+\text{SPDE} \end{array}$$

**TABLE 3:** Model selection metrics for the base model formulation and its extensions for predicting flapper skate presence across the NE Atlantic.

ID	Model formulation	Link function	Dispersion	AICc	BIC	DIC	WAIC	Log mean CV	MSPE
A	Bernoulli GAM	logit	0.561	5487.300	5646.305	4898.595	4897.772	0.186	0.057
B	Bernoulli GAM + smoothed bathymetry	logit	0.567	5519.834	5733.865	4854.025	4853.124	0.184	0.057
C	Bernoulli GAM + smoothed bathymetry + SRF	logit	0.431	5368.182	6122.228	4484.312	4479.245	0.170	0.053
D	Bernoulli GAM + smoothed bathymetry + SRF + cloglog	cloglog	0.430	5371.706	6160.574	4474.495	4471.540	0.170	0.054

*Note:* Model extensions include a random walk smoother for distance to coast, the spatial random field (SRF), and the Complementary log–log (cloglog) link function. The final selected model is highlighted in grey. Metrics reported include AICc (corrected Akaike Information Criterion), BIC (Bayesian Information Criterion), DIC (Deviance Information Criterion), WAIC (Watanabe–Akaike Information Criterion), log mean CV score (cross‐validation score) and MSPE (mean square predictive error).

### 3.1 Model Validation

The final model achieved AUC scores of 0.93 (ROC) and 0.57 (PR), indicating good discrimination of presences for a low prevalence dataset (Figure S6). Sensitivity and specificity were scored as 0.89 and 0.82. The independent simulation study showed that the model predictions were close to those of the observed data, with no significant difference in zero‐inflation observed (z‐score: 0.188; Figure S7). A plateau in the semi‐variogram of spatial residuals showed that the SPDE effect captured autocorrelation in the data well (Figure S8). Finally, the dispersion statistic was 0.43, indicating slight under‐dispersion in the model.

### 3.2 Model Results

**FIGURE 2:**
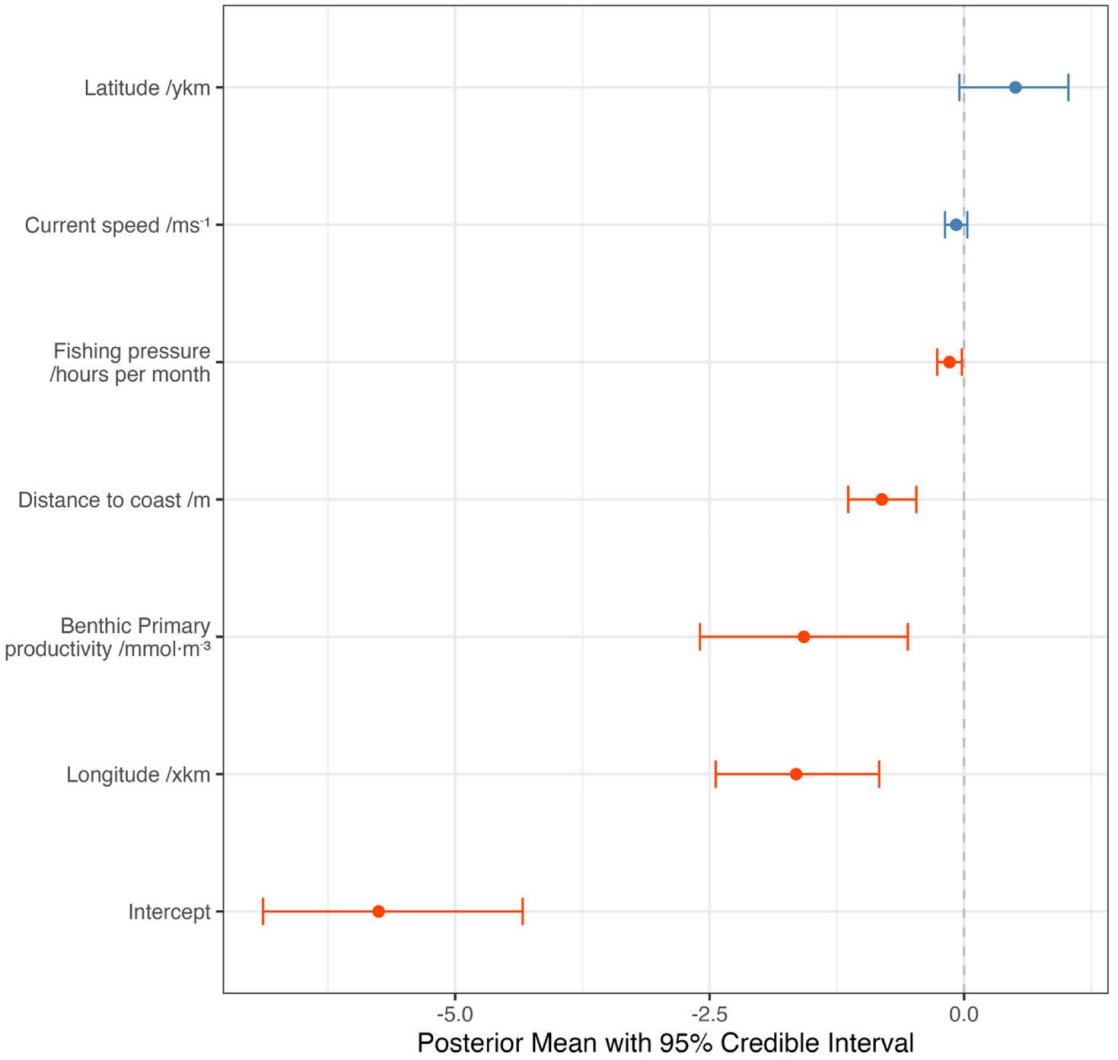
Forest plot of posterior mean estimates and 95% credible intervals for fixed effects in a binomial spatial GAMM of flapper skate presence across the NE Atlantic. Important variables are given in red. Model based on fisheries‐independent catch records extracted from the DATRAS database for the years 2010–2023.

**FIGURE 3:**
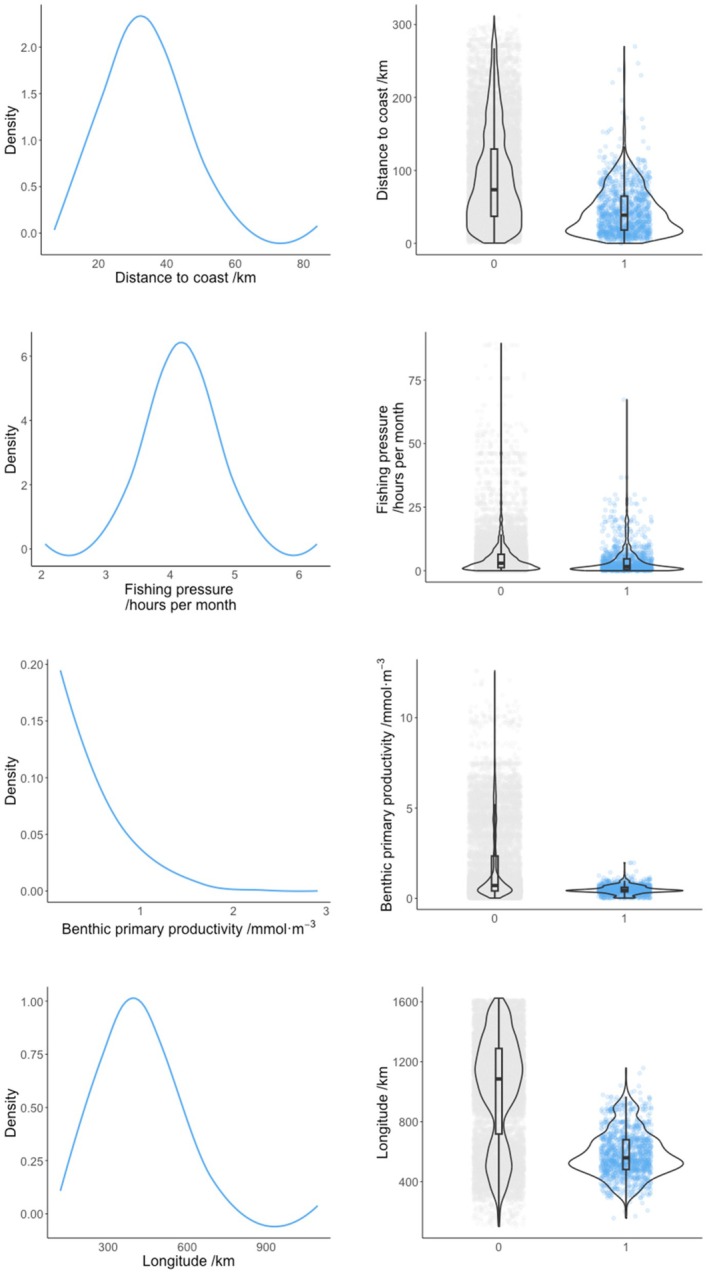
LEFT: Posterior distributions of important fixed effects in a binomial spatial GAMM of flapper skate presence across the NE Atlantic. RIGHT: Violin plot of species presences (1) and absences (0) in relation to each variable. Model based on fisheries‐independent catch records extracted from the DATRAS database for the years 2010–2023.

**FIGURE 4:**
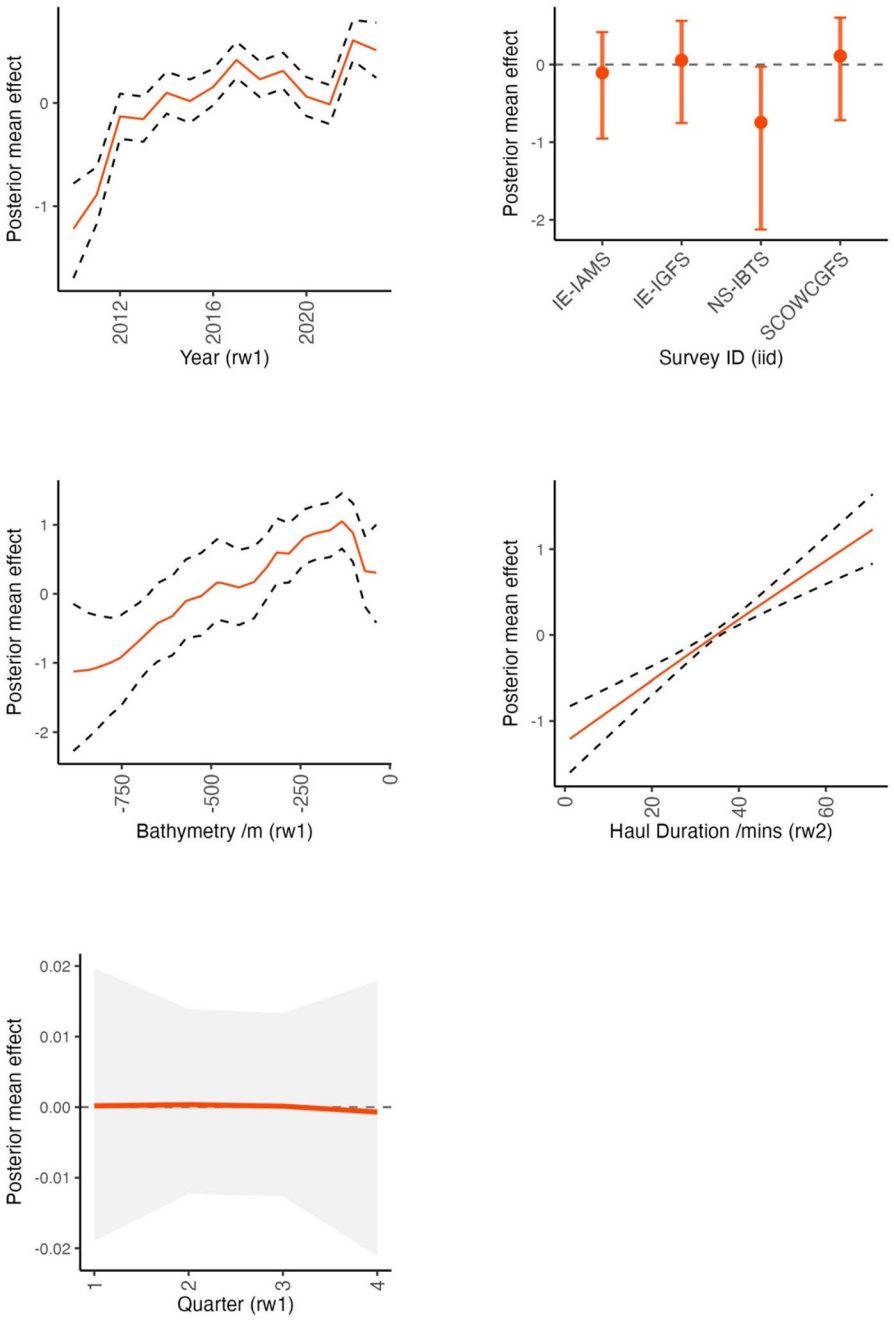
Plot of random effects from the binomial spatial GAMM of flapper presence across the NE Atlantic region, including the estimated effects for the random walk smoothers (rw1 and rw2) and the independent identically distributed (iid) group effects with 95% credibility intervals. Model based on fisheries‐independent catch records extracted from the DATRAS database for the years 2010–2023. IE‐IAMS: Irish Anglerfish and Megrim Survey, IE‐IGFS: Irish Groundfish Survey, NS‐IBTS: The North Sea International Bottom Trawl Survey, SCOWCGFS: Scottish west coast Groundfish Survey.

Distance to coast, fishing pressure, benthic primary productivity and longitude emerged as important drivers of skate presence across the study area (Figure 2). The posterior distributions showed that skate presence was strongly associated with 40–50 km distance from coast, ~ 4.5 h per month fishing pressure, areas of low benthic productivity and in westerly parts of the study area (Figure 3). The smoothed terms indicated an increased trend in skate presence with haul duration and at 100–200 m depth (Figure 4). The smoothed year variable showed an increasing trend in skate presence over time and lower presence in the NS‐IBTS survey compared with other surveys. Skate presence showed minimal changes across quarters. A plot of the spatial random field indicated distinct spatial structuring across the study area due to latent variation (Figure S9).

**FIGURE 5:**
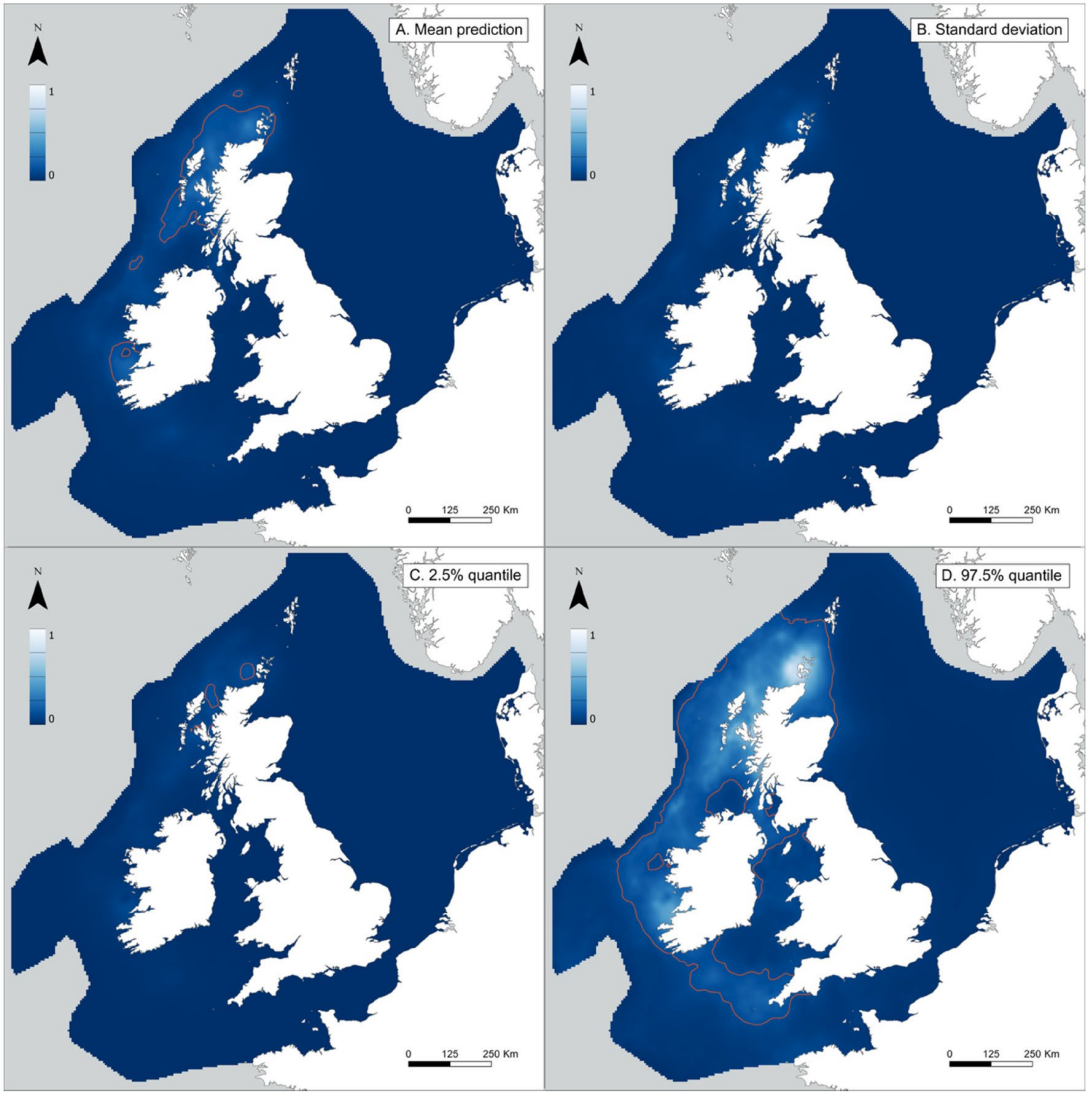
(A) Posterior predictive mean, (B) Standard deviation, (C) 2.5% quantile and 5D: 97.5% quantile of the presence of *D. intermedius* , modelled using fisheries‐independent survey data from the Irish Anglerfish and Megrim Survey (IE‐IAMS), the Irish Groundfish Survey (IE‐IGFS), the Scottish west coast Groundfish Survey (SCOWCGFS) and the North Sea International Bottom Trawl Survey (NS‐IBTS), for the years 2010–2023. The red contour line represents the 0.1 binomial classification threshold used to delineate likely presence of the species.

Areas predicted to have a high probability of skate presence included most prominently the north and west coasts of Scotland (including the Orkney islands), and the western coast of Ireland near county Clare and county Galway (Figure 5). Some support for skate presence was given in the Celtic Sea, and no support was given for the southern and central North Sea region, as well as in deeper waters off the NE Atlantic shelf.

## 4 Discussion

This study aimed to establish baseline information on the flapper skate's distribution across the NE Atlantic to inform future research and management of the species. Following an exhaustive investigation of DATRAS survey data in the region, skate presence was modelled in Irish waters, Scottish waters and in the North Sea, building on previous research for the species.

Model predictions revealed three core areas of flapper skate presence in the NE Atlantic: the north and west coasts of Scotland, and the west coast of Ireland. Information from genetic samples, recreational angling records and commercial fisheries data also support a strong coastal presence of skate in these areas (e.g., Pinto et al. 2016; Frost et al. 2020; Bache‐Jeffreys et al. 2021; Ellis et al. 2021; McGeady et al. 2022; Garbett et al. 2023; Régnier et al. 2024). In contrast, the central and southern North Sea, as well as in deeper waters off the NE Atlantic shelf showed minimal skate presence, reflecting a high number of absences recorded in those areas. Whilst previous studies gave stronger support for skate presence in the central North Sea (Bache‐Jeffreys et al. 2021; Garbett et al. 2023), they employed presence‐only datasets which can inflate predictions of species presence in areas where the sample number is low (Hernandez et al. 2006). These findings clearly show that since its disappearance from the region in the 1970's, the flapper skate's recovery in the southern North Sea has been minimal (Walker and Heessen 1996; Walker and Hislop 1998; Sguotti et al. 2016; Bom et al. 2022).

Skate presence was driven by distance from coast, benthic productivity and fishing pressure; in other words, the core areas are likely acting as natural refugia for the species, where low productivity leads to limited fishing activity, offering relative protection for remnant populations. While previous research also found the flapper skate to be a coastal species of broad habitat requirements (Pinto et al. 2016; Frost et al. 2020; Bache‐Jeffreys et al. 2021; Thorburn et al. 2021; Régnier et al. 2024), this study is the first to integrate environmental information with fishing pressure data across the entire range, providing a more comprehensive picture of the species ecology. Consistent with earlier work (Garbett et al. 2023), our findings point to a fragmented contemporary range shaped by a history of severe depletion and population collapse.

In addition to natural refugia, it is likely that skate presence is related to the presence of critical habitats. Critical or essential fish habitats (EFHs) are areas that are vital for supporting key life stages of a species, including spawning, breeding, feeding and growth to maturity (EFH; U.S. Congress 1996). The use of such habitats is a common motif amongst skate and rays (superorder *Batoidea*; Ellis et al. 2005; Serena and Relini 2006; Serra‐Pereira et al. 2014; Martins et al. 2018; Elliott et al. 2020; McAllister et al. 2024) and examples include juvenile nurseries or egg‐case nurseries (Heupel et al. 2007; Martins et al. 2018). Although little is known of the critical habitats utilised by the flapper skate, one egg‐case nursery has been identified on a shallow cobble/boulder reef off the west coast of Scotland (Dodd et al. 2022). The presence of this site, along with substantial egg‐case sightings around the Orkney Islands and off the west coast of Ireland (Ellis et al. 2024; Phillips et al. 2021), supports the hypothesis that the core areas identified in this study are underpinned by essential habitats for flapper skate.

### 4.1 Temporal Trends in Skate Presence

A positive temporal trend in skate presence was observed in the DATRAS survey data, which mirrors observed trends for the ‘common skate’ species complex (Dulvy et al. 2006; Bom et al. 2022; Régnier et al. 2021; McGeady et al. 2022). Whilst suggestive of a potential recovery trajectory, these data were not corrected for biases in the reportage of flapper skate across the time series (Robinson et al. 2018; Zeller and Pauly 2018). The creation of morphological keys in 2010 to help distinguish blue and flapper skates (Iglésias et al. 2010), as well as the flapper skate's formal recognition in the following years (Ellis et al. 2021), have no doubt influenced detection rates. Therefore, it is likely that the observed ‘increase’ in catch records for flapper skate reflect a concomitant increase in reportage, rather than a recovery to fisheries management measures (Zeller and Pauly 2018). This is further evidenced by the fact that it can take many decades to see population responses to conservation measures in elasmobranchs (Ward‐Paige et al. 2012).

### 4.2 Data Limitations

Although exact incidences of misidentification were hard to prove, existing research has shown that the flapper skate's distribution overlaps with other *Dipturus* skate within the NE Atlantic, including the common blue skate ( *Dipturus batis* ; Frost et al. 2020; McGeady et al. 2022). In some cases, species misidentification can lead to overestimates or underestimates of species presence in certain locations (Beerkircher et al. 2009; Iglésias et al. 2010; Garcia‐Vazquez et al. 2012; Wang 2021). These limitations notwithstanding, the survey data presented here are an important resource for conservation managers and are often the only data available for some species, especially over a long time series (Hamilton et al. 2015; Maes et al. 2015; Jubinville et al. 2021; Min et al. 2023). By employing genetic sampling alongside these surveys, the quality of the data can be improved (see Kinoshita et al. 2019; Koda et al. 2023). Models constructed using these validated species records would provide reliable predictions across a wide study area and could provide validated information on smaller size classes of flapper skate.

Although fishing pressure emerged as a key factor influencing skate presence in the NE Atlantic, the effect size was modest, likely due to limitations in the AIS dataset (Global Fishing Watch 2025b). This dataset predominantly captures activity from larger vessels, underrepresenting smaller inshore boats less than 12 m that are prevalent in coastal fisheries. Moreover, AIS coverage is imperfect, with potential signal gaps caused by limited satellite reception, interference in densely trafficked areas and inconsistencies in transmission strength. Despite these limitations, the fact that skate presence was influenced by this partial measure of fishing activity underscores the species' sensitivity to exploitation, reinforcing previous findings that even moderate levels of fishing pressure can shape the distribution of flapper skate (Brander 1981; Dulvy et al. 2000; Dulvy and Reynolds 2002; Régnier et al. 2021).

### 4.3 Conservation of the Flapper Skate

The flapper skate was once described as a fish on the brink of extinction (Brander 1981), and four decades later the situation has shown little improvement (Garbett et al. 2021, 2023; Ellis et al. 2021; Régnier et al. 2024). The present state of the flapper skate is that of a species relegated to a few natural refugia, with limited evidence of a wider recovery (Garbett et al. 2023; Régnier et al. 2024; this study). The observed restricted distribution is likely a direct consequence of continued fishing pressure, as individuals across all life stages remain exposed throughout much of the species' range (e.g., see catch records from Iglésias et al. 2010; Frost et al. 2020; Régnier et al. 2024; this study). This ongoing pressure is particularly concerning given the species' extreme life history vulnerability, which renders it unable to recover from even moderate depletion. As a Critically Endangered species now confined to the margins of its historical range, the flapper skate is at imminent risk of further decline without the implementation of a more robust and spatially comprehensive conservation strategy. In light of its recognised conservation value across UK and European waters (HELCOM 2006; OSPAR Commission 2008; Clarke et al. 2016; NatureScot 2020; Ellis et al. 2021), member states must act without delay to close existing protection gaps, prioritising the safeguarding of juveniles and the preservation of critical habitats to ensure the species' long‐term survival.

This study presents the first large‐scale model of flapper skate presence across the NE Atlantic shelf that integrates both environmental and fishing pressure data, and provides new baseline information on skate habitat use in the North Sea and around the island of Ireland. The results identify three core areas of presence in the NE Atlantic shelf that appear to function as natural refugia, shaped by low fishing pressure and potentially underpinned by critical habitats. Future research should prioritize these remaining strongholds for the species, focusing on the identification and protection of critical habitats to guide effective spatial management strategies.

## Author Contributions

**Sophie L. Loca:** conceptualization (equal), formal analysis (lead), investigation (lead), methodology (lead), visualization (lead), writing – original draft (lead), writing – review and editing (lead). **Patrick C. Collins:** conceptualization (equal), funding acquisition (lead), project administration (lead), supervision (lead), writing – original draft (supporting), writing – review and editing (supporting). **Ryan McGeady:** methodology (supporting), writing – review and editing (supporting). **Amy Garbett:** writing – original draft (supporting). **James Thorburn:** writing – original draft (supporting). **Chris McGonigle:** methodology (supporting), supervision (supporting), writing – original draft (supporting).

## Conflicts of Interest

The authors declare no conflicts of interest.

## Supporting information


Appendix S1.

## Data Availability

All R scripts and datasets used in the analysis are available online at: https://doi.org/10.5061/dryad.w0vt4b954.
