# Supplementary material for: On the Brink: Mapping the Last Strongholds of the Critically Endangered Flapper Skate (Dipturus intermedius)
Source: Ecol Evol. 2025 Jun 30;15(7):e71650. doi: 10.1002/ece3.71650 (PMC12209335; doi:10.1002/ece3.71650)
Supplement: Supplementary file 1 — Appendix S1. [file ECE3-15-e71650-s001.docx]

**Appendix**

Table S1. Generalised Variance Inflation Factors (GVIFs) of continuous explanatory variables in the flapper skate occurrence dataset.

| **Variable** | **GVIF** |
| --- | --- |
| Bathymetry /m | 1.556 |
| Bottom Temperature /°C | 2.375 |
| Current speed /ms-1 | 1.091 |
| Distance to Coast /m | 1.594 |
| Fishing pressure /hours per month | 1.044 |
| Haul Duration /mins | 1.286 |
| Benthic Primary Productivity /mmol. m-3 | 2.155 |

Table S2. Classification thresholds tested for a binomial model of flapper skate presence across the NE Atlantic shelf. SSS: sum of sensitivity and specificity.

| **Threshold** | **Sensitivity** | **Specificity** | **SSS** |
| --- | --- | --- | --- |
| 0.1 | 0.887 | 0.822 | 1.708 |
| 0.2 | 0.723 | 0.910 | 1.633 |
| 0.3 | 0.578 | 0.954 | 1.533 |
| 0.4 | 0.408 | 0.978 | 1.386 |
| 0.5 | 0.200 | 0.993 | 1.193 |
| 0.6 | 0.075 | 0.999 | 1.074 |
| 0.7 | 0.018 | 1.000 | 1.018 |
| 0.8 | 0.001 | 1.000 | 1.001 |

**
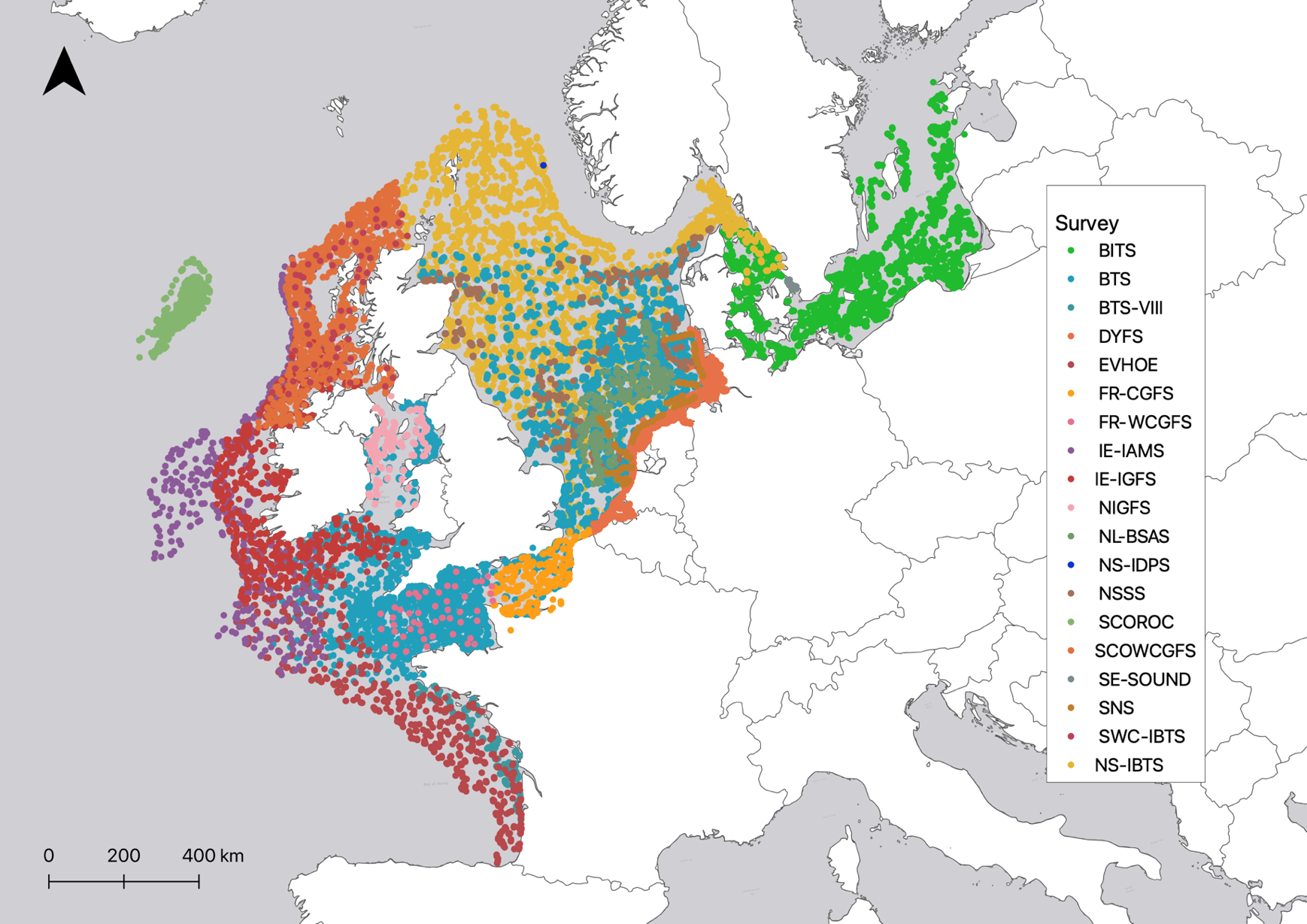
**

Figure S1. Map of DATRAS fisheries-independent surveys for the NE Atlantic Shelf Region. Data were extracted for the years 2010-2023.

Figure S2. Study of correlations between explanatory variables in the flapper skate occurrence dataset using Pearson’s rank correlations. Negative correlations between variables are represented by red ellipses and positive correlations are represented by blue ellipses. When two variables were shown to be highly correlated the ellipse thickness was thin.

**
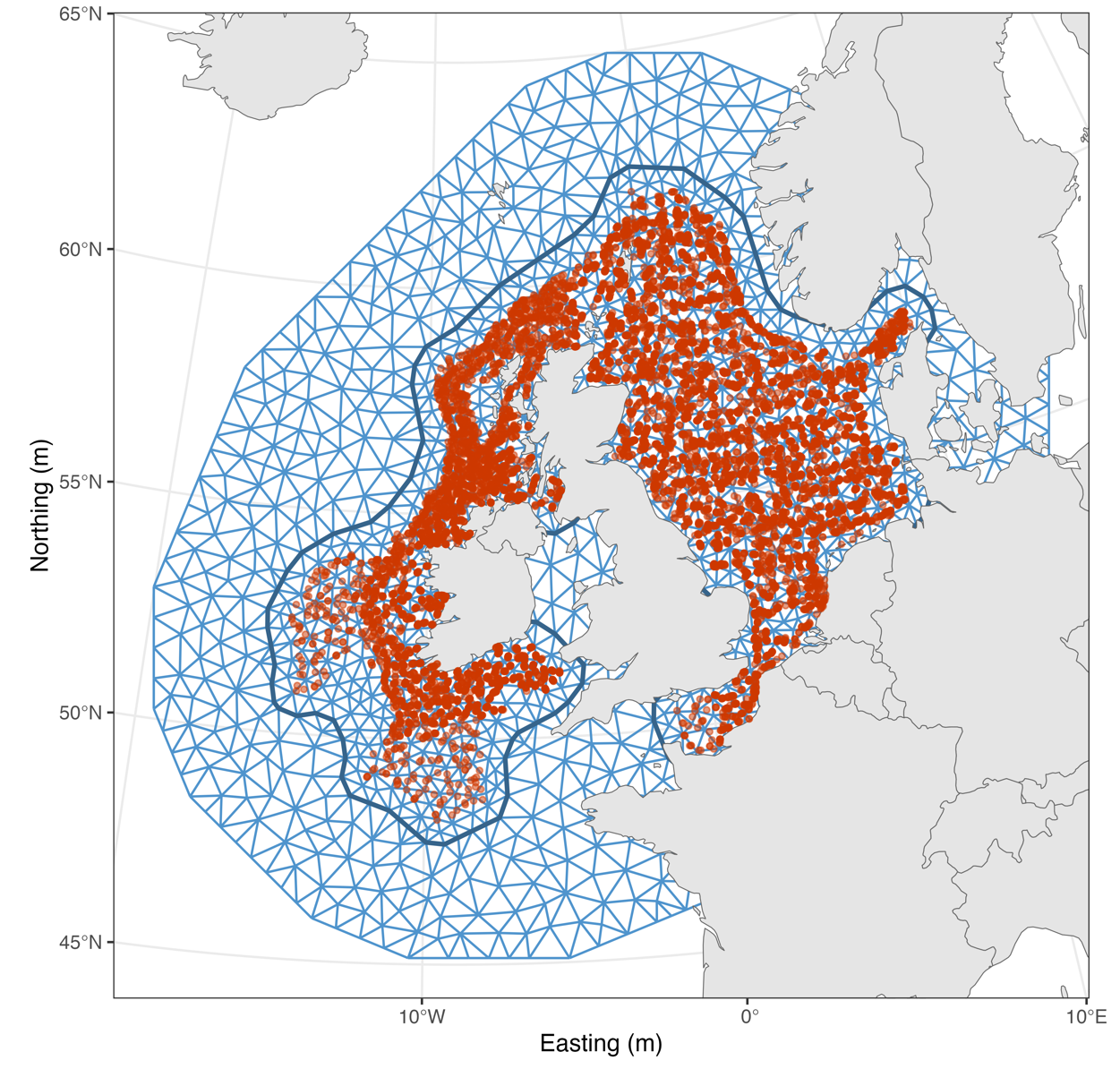
**

Figure S3. Stochastic Partial Differential Equation triangulated mesh generated to represent the spatial random field in a binomial GAMM of flapper skate presence across the NE Atlantic (n= 1615). Red dots indicate haul locations and the dark blue line represents the inner boundary of the random field.


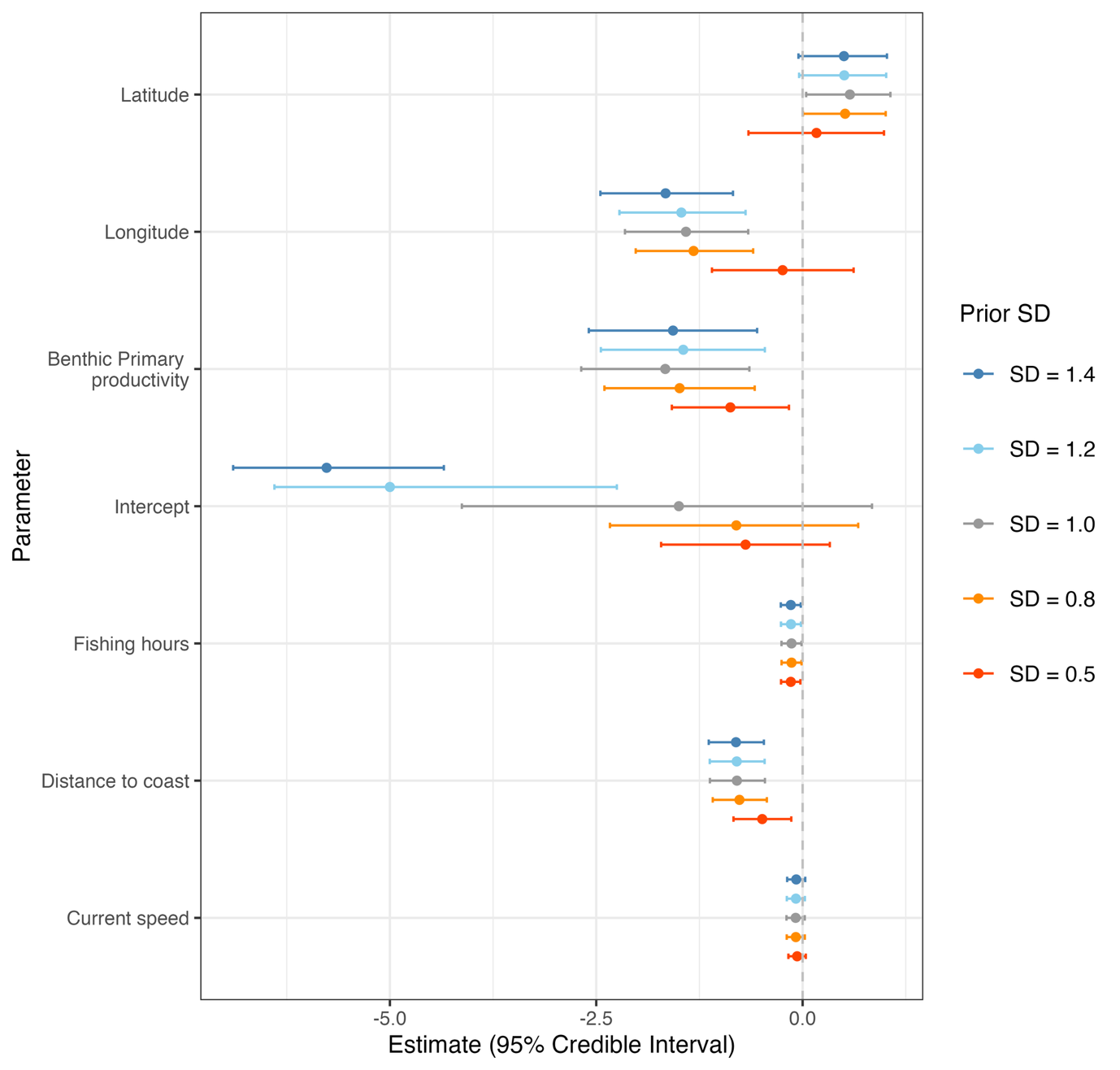


Figure S4. Forest plot of posterior mean estimates and 95% credible intervals for fixed effects under different fixed prior standard deviations (SDs). A prior with SD = 1.4 is considered “weakly informative”.

**
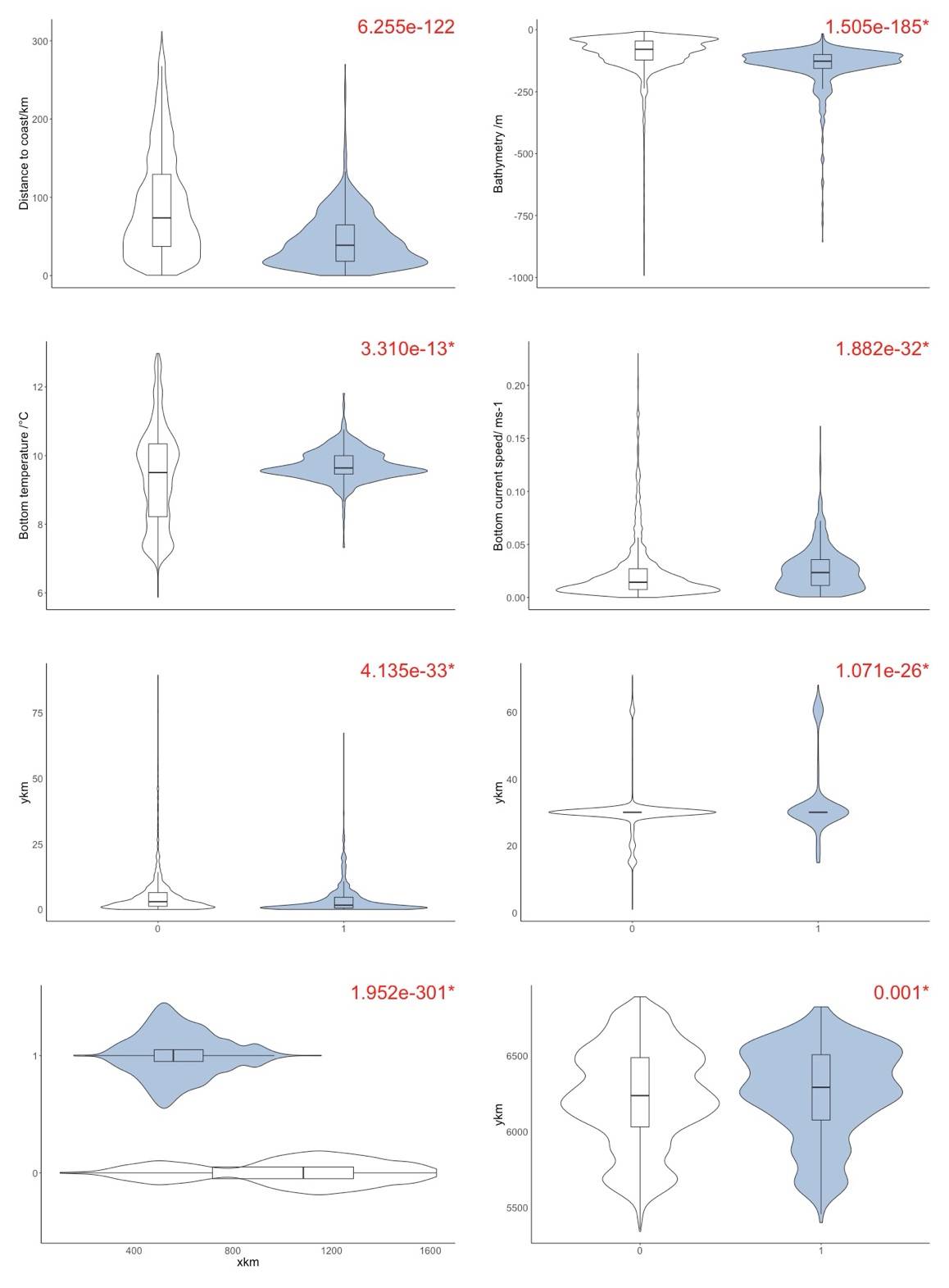
**

Figure S5. Presence and absence locations of flapper skate in relation to environmental variables. Skate presence and absence per haul was extracted from the DATRAS data portal, for the years 2010-2023. Statistical differences between presence and absence hauls were investigated using unpaired Wilcoxon rank sum tests. P-values are indicated above each plot, and significant values where p < 0.05 are coloured red and indicated with a star.


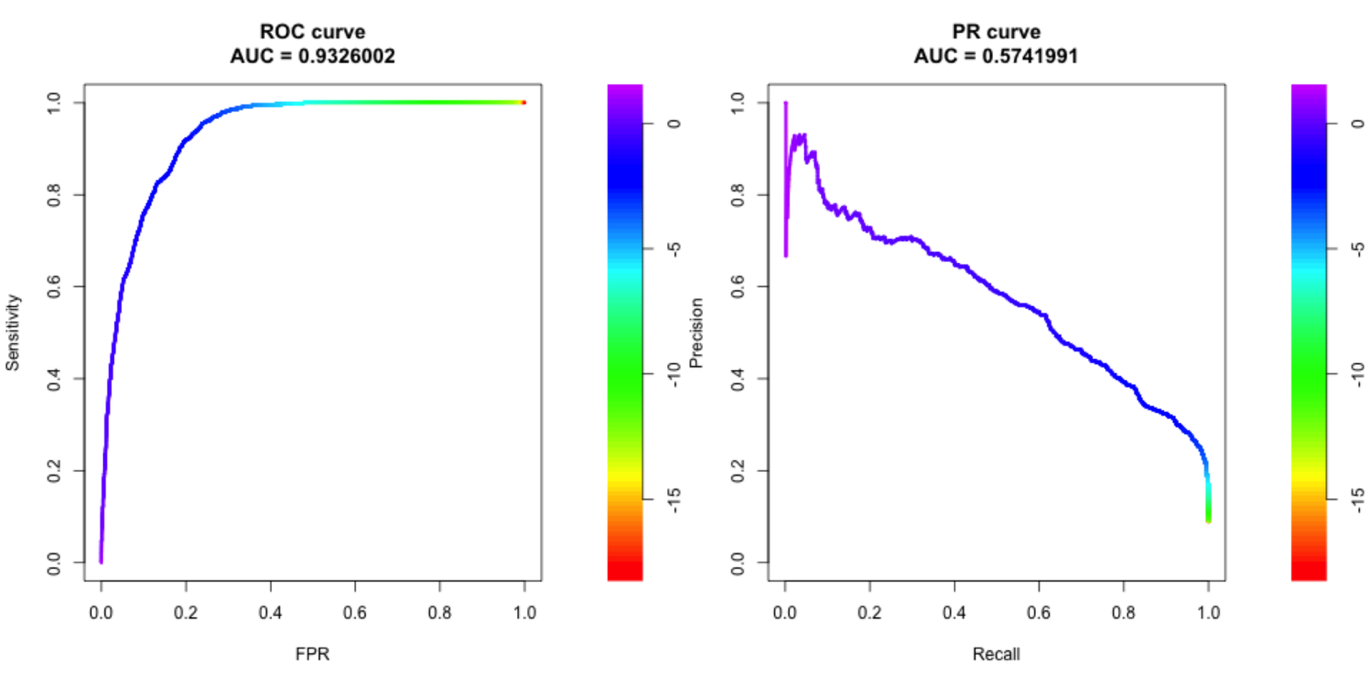


Figure S6. Receiver Operating Characteristic (ROC) and Precision Recall (PR) curves for the final model (model D) of flapper skate presence, generated using Leave-One-Group-Out Cross-Validation (LGOCV). The colour scale represents the classification threshold.


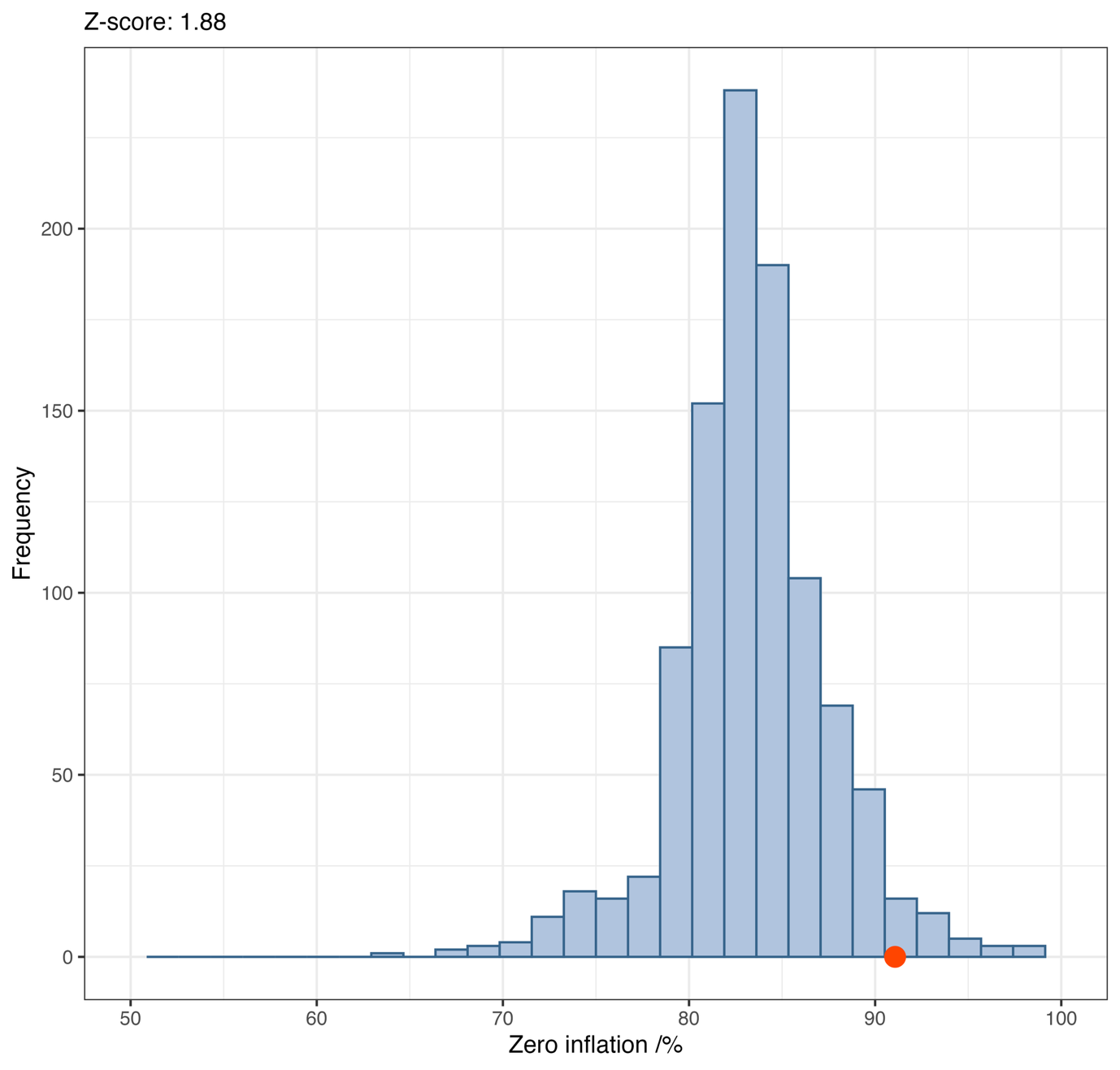


Figure S7. Simulation study carried out on a binomial spatial GAMM of flapper skate presence in the NE Atlantic. The predictive ability of the models was tested by comparing the proportion of zeros produced from 1000 simulated datasets with the observed data. The red dot represents the observed data**.**


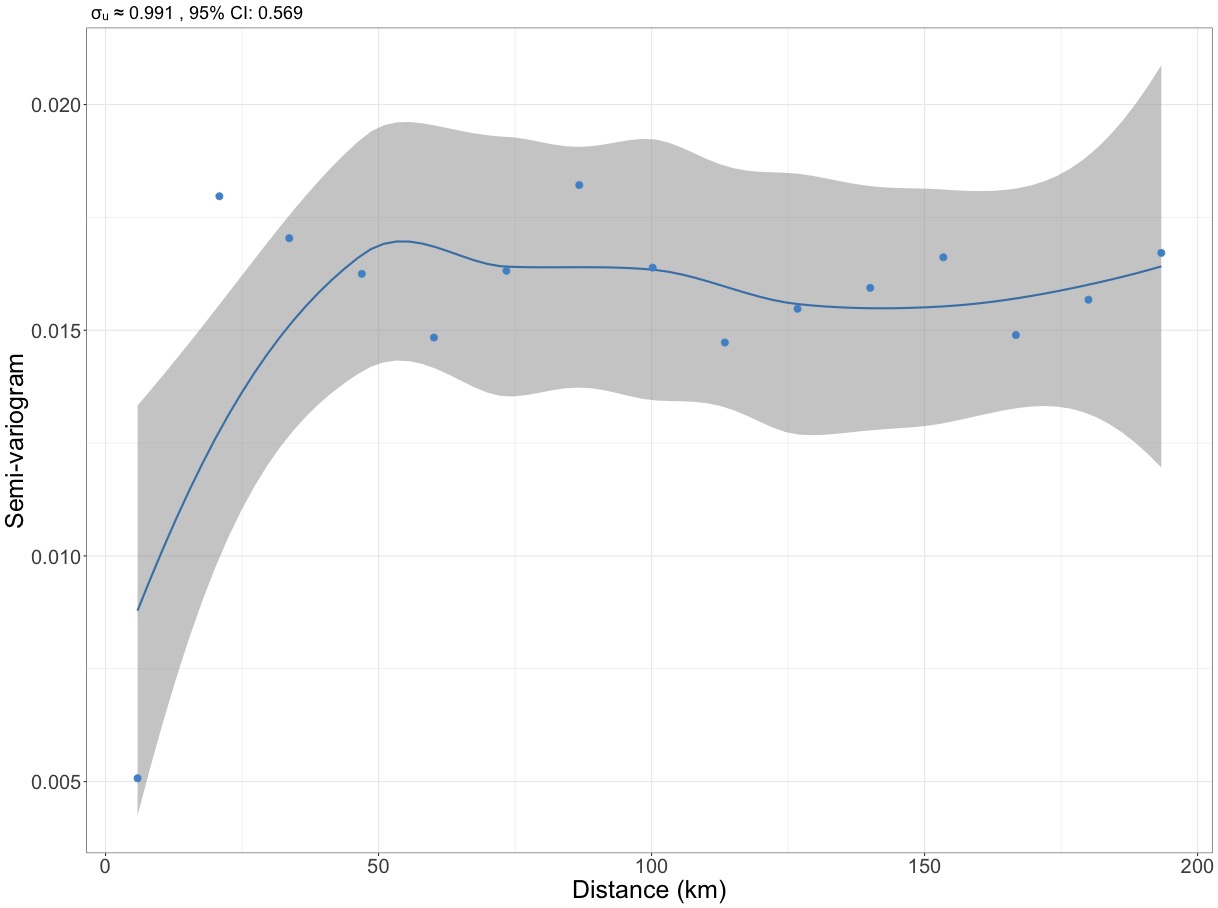


Figure S8. Semi-variogram of spatial residuals in a binomial spatial GAMM of flapper skate presence across the NE Atlantic.

**
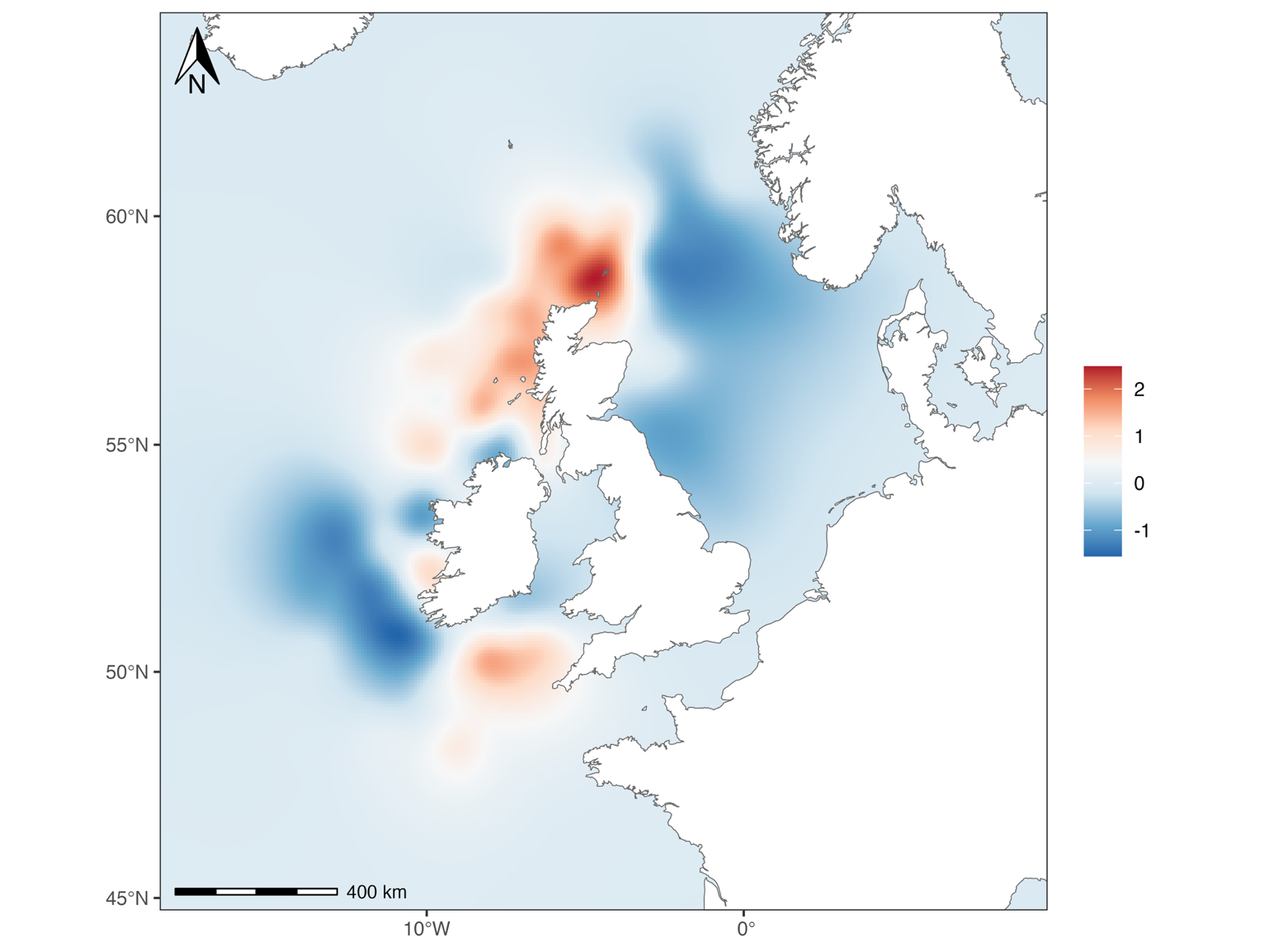
**

Figure S9. Map of the spatial random field in a binomial spatial GAMM of flapper skate presence across the NE Atlantic. Modelled using the R-INLA SPDE approach.
